# Myocardial rupture after percutaneous coronary intervention of an unstable RCA lesion in myocardial infarction and concomitant stroke treated with intravenous fibrinolytic agents: A case report

**DOI:** 10.1002/ccr3.3736

**Published:** 2021-01-08

**Authors:** Benjamin Bay, Christoph Waldeyer, Leander Rimmele, Stefan Blankenberg, Peter Clemmensen

**Affiliations:** ^1^ Department of Cardiology University Heart & Vascular Center University Medical Center Hamburg Hamburg Germany; ^2^ Department of Neurology University Medical Center Hamburg Hamburg Germany

**Keywords:** myocardial infarction, myocardial rupture, peri‐interventional stroke, thrombectomy, thrombolysis

## Abstract

Myocardial rupture after thrombolysis is an often fatal consequence. For patients with myocardial infarction and ischemic stroke within a short timeframe, a catheter‐based therapy to retrieve emboli poses a valid therapeutic option in case of a treatment target in CT‐Angiography.

## Graphical abstract

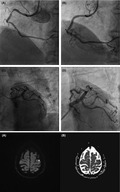

## 1 INTRODUCTION

We present a case of myocardial rupture with fatal outcome shortly after percutaneous coronary intervention (PCI) for an unstable Non–ST‐Segment Myocardial Infarction (NSTEMI) followed by an ischemic stroke treated with intravenous (iv) Alteplase.

Myocardial rupture is a rare, but often fatal, complication after myocardial infarction. Intravenous fibrinolytic therapy given late in the setting of myocardial infarction is associated with an increased risk of hemorrhagic necrosis and myocardial rupture. Due to the development of primary percutaneous coronary interventions for myocardial infarction instead of intravenous fibrinolysis, a dramatic decrease in regard to mortality has been registered, mainly due to less myocardial re‐infarction. Patients with a recent myocardial infarction and subsequent stroke present a dilemma to the treating physicians due to the increased potential of intramyocardial hemorrhage and myocardial perforation after iv thrombolysis for ischemic stroke.

In this article, we present the case of a 79‐year‐old patient with myocardial rupture and following fatal outcome after iv thrombolysis applied for a stroke in the setting of emergent PCI for an unstable proximal RCA lesion.

**FIGURE 1:**
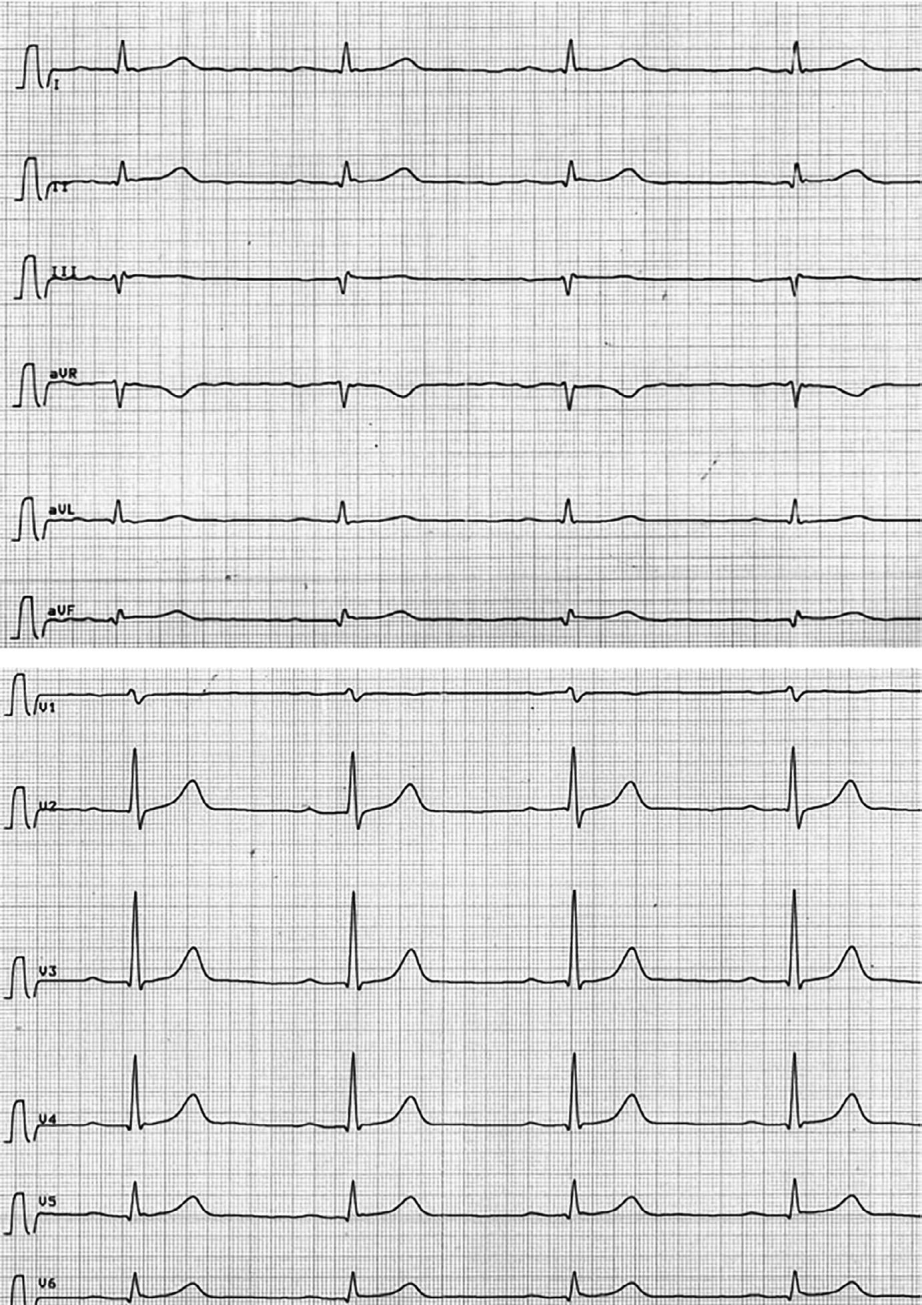
Electrocardiogram with nonsignificant ST Elevation in leads II, III, aVF, and V6

Perforation of the myocardium after iv thrombolysis presented a common challenge during the era of fibrinolytic therapies applied for myocardial infarction. Current guidelines regarding early causative therapy for ischemic stroke do not view a recent myocardial infarction as a contraindication for iv thrombolysis. The concomitant occurrence of myocardial infarction and ischemic stroke poses a challenge to the treating Heart and Brain team. Due to the high mortality of myocardial rupture when iv lysis is given later than 12‐24 hours after an acute MI, the interdisciplinary discussion should consider mechanical thrombectomy instead of lysis when feasible.

**FIGURE 2:**
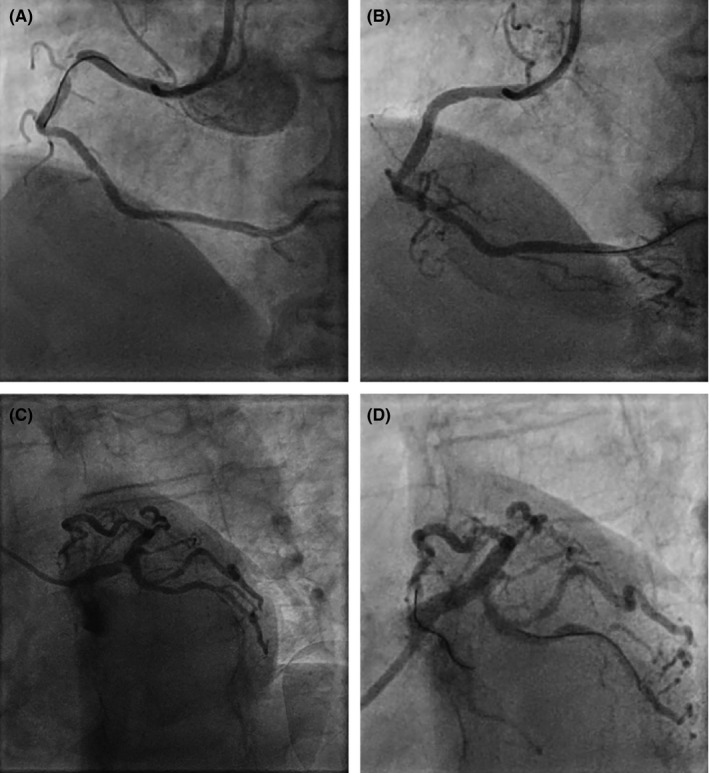
RCA Lesion before (A) and after (B) PCI, LM/LAD Lesion before (C) and after (D) PCI

## 2 CASE HISTORY

A 79‐year‐old male patient with no prior cardiac diagnosis presented to a local hospital on the 14th January due to acute onset of angina pectoris after physical exertion (complete timeline is displayed in Table 1). The initial electrocardiogram displayed a sinus rhythm with borderline ST‐segment elevation in leads II, III, and aVF (Figure 1). The initial high sensitive Troponin T (hsTnT) levels were recorded at a 58 pg/mL, which after 3 hours rose to 161 pg/mL. The further laboratory results were unremarkable. Echocardiography showed no abnormalities.

**TABLE 1:** Timeline

Date	Event
January 14, 2020	Patient presents to ED with angina pectoris ECG: SR, nonsignificant ST Elevation initial hsTnT 58 pg/mL, CK 121 U/L. After 3 h hsTnT 161 pg/mL Echocardiography: normal ventricular functions, grade 1 mitral valve incompetence Coronary angiography: three vessel coronary artery disease with involvement of the left main coronary artery (MLA of 3.1 mm^2^), a proximal LAD stenosis and a tight middle RCA stenosis, considered the culprit. Transfer to University clinic for further treatment evaluation
January 15, 2020	01:00 am: nsVT for 30 s, patient hemodynamically unstable with elevation of lactate and mild hypokalemia 08:00 am: rising hsTnT 423 pg/mL; CK 276 U/L, 18% CK‐MB 12:00 am: PCI of the unstable RCA lesion with one DES
January 17, 2020	05:00 pm: PCI of the LM and LAD with two DES 07.30 pm: aphasia, no motoric involvement 08.30 pm: cCT and CT‐Angiography without abnormalities, 09.15 pm: cMRI with demarcation of left precentral gyrus 09.45 pm: initiation of iv fibrinolytic therapy with Alteplase
January 18, 2020	00:30 am: bradycardia on telemetry followed by asystole. Echocardiography without pericardial effusion. CPR for 30 min, no ROSC 01:00 am: Exitus letalis

Previous illnesses of the patient included an intracerebral hemorrhage 11 years prior, a transient ischemic attack (TIA), and known localized cancer of the prostate (in remission) for which the patient had been treated with radiotherapy. Regarding cardiac risk factors, the patient presented with a known arterial hypertension and hypercholesterinemia. At time of admission, the patient was receiving statin treatment (simvastatin 40 mg/day), antihypertensive therapy (ramipril/HCT 5/12.5 mg/day; bisoprolol 5 mg/day) and was also taking a platelet aggregation inhibitor (aspirin 100 mg/day).

Acute coronary angiography was carried out via a radial approach, in which a three vessel coronary artery disease (CAD) was documented due to stenoses of the Left main (LM), proximal Left anterior descending (LAD), and mid‐Right coronary artery (RCA). An intravascular ultrasound (IVUS) study of the LM showed a minimum lumen area (MLA) of 3.1 mm^2^, thus a highly significant stenosis. Due to LM involvement, the patient was transferred to our tertiary center on the 14th January for further evaluation and possible subacute coronary artery bypass graft (CABG). The use of cardiopulmonary bypass machine was deemed possible despite the previously described, albeit distant intracerebral hemorrhage, however, with increased risk of bleeding complications.

**FIGURE 3:**
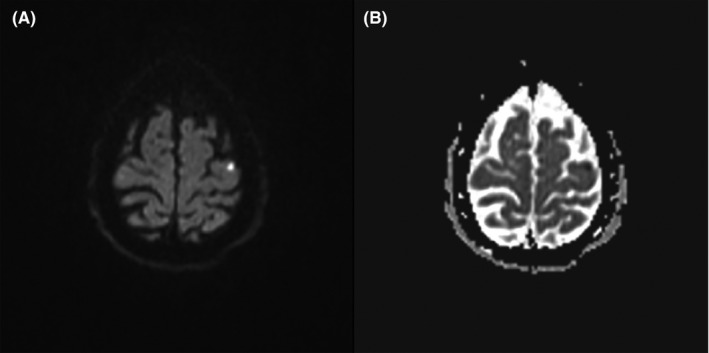
cMRI with a small corticomedullary diffusion restriction (A) and with correlating signal reduction in the displayed apparent diffusion coefficient (ADC) map (B)

During continuous monitoring, a nonsustained ventricular tachycardia for 30 seconds was registered. Blood gas analysis showed a transient elevation of lactate. The next morning, the hsTnT rose further from 201 at admission to 423 pg/mL and elevated creatine kinase (CK) was detected (276 U/L) with a relevant (18%) proportion of CK‐MB. Due to hemodynamic destabilization with progressive hypotension, immediately an ad hoc heart team reversed the strategy with the recommendation to intervene with PCI targeting the unstable RCA lesion. In addition to the existing aspirin therapy, loading with a P2Y12 inhibitor occurred with 600 mg of clopidogrel. On the 15th, the RCA as culprit lesion (Figure 2 A and B) was treated by percutaneous coronary intervention (PCI) with one drug‐eluting stent (DES, PROMUS 3.0 × 20 mm). Two days later, on the 17th, the LM and LAD (Figure 2 C and D) were also treated by PCI (LM ‐ PROMUS DES 3.5 × 16 mm, LAD ‐ PROMUS DES 3.0 × 12 mm). Adequate positioning of the DES was controlled by IVUS. During the LM intervention 8.000 International units (IU) of heparin were administered (106 IU/kilogram body weight). The course of the intervention (Contrast load of 138 mL, fluoroscopy time 17.2 minutes) and the immediate monitoring hereafter was uneventful.

After transfer to the monitoring ward one hour after the PCI, the patient developed aphasia without any motor deficit. Initial neurological assessment showed a score of 7 on the National Institutes of Health Stroke Scale (NIHSS) and a score of 0 on the modified Rankin Scale (mRS). Further imaging studies were initiated immediately due to the working diagnosis of a peri‐interventional stroke. In the cranial computed tomography (cCT) and angiography (CT‐A), no acute intracerebral hemorrhage, ischemic tissue demarcation, or occluded cerebral artery was registered. Because of the persisting symptoms, a magnetic resonance imaging of the neurocranium (cMRI) with diffusion‐weighted imaging (DWI) was carried out. Due to the demarcation of the left precentral gyrus in the DWI analysis (Figure 3), persisting aphasia, and a NIHSS Score of 7 an intravenous fibrinolytic therapy with Alteplase (Patient weight 75 kg, Bolus of 6.8 mg, continuous infusion of 61.2 mg over 1 hour hereafter) was initiated from 10 pm onwards on the 17th. The patient was then transferred to the stroke unit for further monitoring. At 0.30 AM on the 18th January, bradycardia followed by asystole was registered on telemetric monitoring. Immediate cardiopulmonary resuscitation (CPR) was carried out; initial echocardiography showed no pericardial effusion. No return of spontaneous circulation (ROSC) could be achieved. After 30 minutes with no ROSC, CPR was terminated.

In the autopsy, the pathologists were able to detect a rupture of the free wall of the right ventricle with acute myocardial hemorrhage and pericardial effusion with 300 mL of serosanginous fluid. The implanted stents showed no obstruction. Studies of the cerebral tissue were able to document no intracerebral bleeding.

## 3 DISCUSSION

The incidence of myocardial rupture with subsequent cardiac tamponade and fatal outcome after intravenous fibrinolytic therapy for MI has been described previously. Before the advent of coronary interventions, iv thrombolysis was the only feasible option to treat patients with an acute MI. Patel et al describe an incidence of 0.85% myocardial perforations in a cohort of more than 100 000 patients treated with iv thrombolysis after ST‐segment elevation myocardial infarction (STEMI).^1^ Increasing age, anterior MI location, female sex, increased time from symptom onset to treatment, which has also been reported by Honan and colleagues, were identified as independent risk factors with an increased risk for myocardial perforation.^2^ The emergence of coronary interventions and the broad availability of catheter laboratories to carry out PCI in case of a MI have seen a stark decrease of iv lysis due to the reduced mortality of patients treated with primary PCI.

The concomitant appearance of MI and stroke remains a dilemma facing the interdisciplinary Heart and Brain team of cardiologists and neurologists. In 2.3% of all patients with an MI, an ischemic stroke can be documented in the first month after myocardial infarction, which in comparison to a control cohort is a 44‐fold increased risk for stroke.^3^

In the updated version of the American Heart Association/American Stroke Association (AHA/ASA) stroke guidelines, in patients with recent MI an iv lysis is considered reasonable.^4^ Marto et al present a case series and review of the literature describing 4 cardiac ruptures in 47 STEMI patients treated with iv fibrinolytic therapy for stroke up to 7 days after the index MI.^5^ Therefore, it is necessary to weigh potential benefits against risks individually. In our case, severe persisting symptoms of disabling aphasia were timely detected, and a mismatch on cerebral imaging suggested a benefit of lysis. On the other hand, risks of complications were increased by the recent myocardial infarction and a history of intracranial hemorrhage. Since 2013, the latter is no longer considered a contraindication, albeit associated with a higher risk of complications. Without signs of microbleeds on the cMRI scan, recent studies show that the risk of secondary bleeding is not increased by thrombolysis.^6^ With this knowledge, and the fact that no vascular target was identified by CT‐A for endovascular recanalization, the decision for thrombolysis was made in our case.

Since the incidence of catheter‐associated strokes is rising due to the number cardiac catheterizations being carried out, this treatment dilemma will become a more frequent challenge.^7^ While the recent application of intravenous heparin is an absolute contraindication for iv lytic therapies, there are national consensus papers that recommend the application of iv or intra‐arterial (ia) fibrinolytic therapies in the setting of catheter‐associated peri‐interventional stroke.^4^, ^8^, ^9^ A feasible option without the need for iv or ia utilization of fibrinolytic agents seems to be a catheter‐based thrombectomy of cerebral emboli in case of a target lesion in cerebral imaging. Systematic studies in regard to a catheter‐based treatment of ischemic stroke after PCI are, to the best of our knowledge, not available. However, due to the small cerebral infarction in our patient, a catheter‐based treatment strategy would have not been suited in this case.

All considered, myocardial rupture after iv thrombolysis is a rare, but often fatal consequence. For patients with MI and ischemic stroke within a short timeframe, no concrete recommendations can be given. A catheter‐based therapy to retrieve emboli that seem to be causative for stroke after coronary angiography poses a valid treatment option and requires further investigation.

## CONFLICT OF INTEREST

None declared.

## AUTHOR CONTRIBUTIONS

BB and PC: involved in conception, literature research, and writing of the case report. CW, DLR, and SB: involved in research and critical revision of the manuscript.

## ETHICAL APPROVAL

Written and informed consent was obtained from the patients next of kin in regard to publication of this article.
